# A critical review of the research literature on Six Sigma, Lean and StuderGroup's Hardwiring Excellence in the United States: the need to demonstrate and communicate the effectiveness of transformation strategies in healthcare

**DOI:** 10.1186/1748-5908-4-35

**Published:** 2009-07-01

**Authors:** Joshua R Vest, Larry D Gamm

**Affiliations:** 1 Department of Health Policy and Management, School of Rural Public Health, Texas A&M Health Science Center, College Station, Texas, USA

## Abstract

**Background:**

U.S. healthcare organizations are confronted with numerous and varied transformational strategies promising improvements along all dimensions of quality and performance. This article examines the peer-reviewed literature from the U.S. for evidence of effectiveness among three current popular transformational strategies: Six Sigma, Lean/Toyota Production System, and Studer's Hardwiring Excellence.

**Methods:**

The English language health, healthcare management, and organizational science literature (up to December 2007) indexed in Medline, Web of Science, ABI/Inform, Cochrane Library, CINAHL, and ERIC was reviewed for studies on the aforementioned transformation strategies in healthcare settings. Articles were included if they: appeared in a peer-reviewed journal; described a specific intervention; were not classified as a pilot study; provided quantitative data; and were not review articles. Nine references on Six Sigma, nine on Lean/Toyota Production System, and one on StuderGroup meet the study's eligibility criteria.

**Results:**

The reviewed studies universally concluded the implementations of these transformation strategies were successful in improving a variety of healthcare related processes and outcomes. Additionally, the existing literature reflects a wide application of these transformation strategies in terms of both settings and problems. However, despite these positive features, the vast majority had methodological limitations that might undermine the validity of the results. Common features included: weak study designs, inappropriate analyses, and failures to rule out alternative hypotheses. Furthermore, frequently absent was any attention to changes in organizational culture or substantial evidence of lasting effects from these efforts.

**Conclusion:**

Despite the current popularity of these strategies, few studies meet the inclusion criteria for this review. Furthermore, each could have been improved substantially in order to ensure the validity of the conclusions, demonstrate sustainability, investigate changes in organizational culture, or even how one strategy interfaced with other concurrent and subsequent transformation efforts. While informative results can be gleaned from less rigorous studies, improved design and analysis can more effectively guide healthcare leaders who are motivated to transform their organizations and convince others of the need to employ such strategies. Demanding more exacting evaluation of projects consultants, or partnerships with health management researchers in academic settings, can support such efforts.

## Background

Growing evidence demonstrates that the American healthcare delivery system falls short of care that is safe, effective, efficient, patient centered, timely, and equitable, as called for by the Institute of Medicine [1]. Although health systems are continually innovating in management and clinical practices, significant and sustained changes will be necessary in most health organizations if crises portended for healthcare are to be averted [2]. Required are transformational changes in health organizations that fundamentally alter practices and culture, and lead to more effective and efficient healthcare.

### Conceptual Framework

Numerous scholars have attached varying definitions to the phrases organizational transformation and transformational changes. For example, King defined organizational transformation as, 'a planned change designed to significantly improve overall organizational performance by changing the behavior of a majority of people in the organization' [3]. Likewise, Levy and Merry wrote '(s)econd-order change (organizational change) is a multidimensional, multi-level, qualitative, discontinuous, radical organizational change involving a paradigmatic shift'[4]. Other words used to describe transformation include: radical, profound, fundamental change, or modification of patterned behavior [5,6]. Transformational interventions disrupt periods of relative equilibrium, in which organizations are entrenched in existing processes, routines, and culture, and only focusing on incremental adjustments [7]. From these revolutions, the organization emerges to a period of new stability with cultural changes [4], and new and improved processes and outcomes [8] that better meets the needs of its customers [5].

Transformation is visionary strategy that is integrated into the organization and then develops the organization's capabilities [5]. Therefore, transformation is a phenomenon beyond simple innovation adoption, or scanning the environment for new knowledge or practice assets. Innovation is frequently identified with a new product or practice that has to do with the production technologies (the methods and processes for transforming inputs into outcomes) of an organization. Adopting and routinizing innovations capable of generating fundamental organization-wide change in practices is a necessary condition of transformation. However, simple innovation routinization is not a sufficient condition, given that definitions of transformation also incorporate shifts of collective behavior or values pointing to organization-wide culture change. Therefore, we view change in practices and change in culture as two essential elements in transformation (see Table 1). The inability of many organizations to ensure transformation along both these dimension may explain a number of previous failings of lauded approaches like process reengineering or continuous quality improvement (CQI) to be viewed by employees and staff as anything different than a passing management fad [9,10].

**Table 1:** Relationship of change and practice.

	No Change in Practices	Transformation in Practices
No Change in Culture	Stasis	Reluctant participants Failed implementation

Transformation in Culture	Turnover, loss of best people	Sustainable organizational transformation

For the purpose of this review, a transformation fundamentally alters both practices and culture, and leads to improved healthcare. For healthcare organizations, practices encompasses activities in the areas of administrative, clinical, social, or information technologies [11]. Technologies being defined as 'tools, devices, and knowledge' [8]. We adopt a planned or orchestrated view of transformation that acknowledges 'uncontrollables' exist, but recognizes the active role of management. Transformation strategies of interest here are managerial practices and approaches directed at changing operations and culture in order to address the Institute of Medicine identified shortcomings of health service organizations.

The field of healthcare management is no stranger to transformational efforts. Efforts like total quality management (TQM) and process reengineering, although pushed by the institutional environment, failed to translate into sustainable results [12]. Likewise, the new organizational forms developed through consolidation, integration, and relationships between hospitals and physician organizations produced a mix of benefits and negatives with many questions left unanswered [13]. Currently, several strategies are endorsed as transformational both in the trade literature and by healthcare leaders who offer convincing 'evidence from practice' that these efforts produce results.

What is the extent to which the evidence for effectiveness is demonstrated in well-structured research and communicated via the peer-reviewed literature for current popular transformation strategies? Likewise, what evidence exists these transformational strategies change both practices and organizational culture? Such research and communication is critical to demonstrating effectiveness, and to providing insights for ensuring proper implementation in the healthcare setting. Accordingly, we reviewed the current healthcare literature, summarized results, and made recommendations for further avenues and modes of research.

## Methods

We selected three transformation strategies for examination: Six Sigma, Lean/Toyota Production System, and StuderGroup's Hardwiring Excellence. This list, however, is by no means exhaustive of all the potential strategies available, but were three strategies identified by the authors as recurrent themes based on a regular attention to health management publications, and through discussions with members of the Southeast Texas Chapter of the American College of Healthcare Executives as currently popular among their colleagues. For example, articles in trade publications have credited both Six Sigma [14,15] and Lean/Toyota Production System [16] with hospital successes. Additionally, StuderGroup's Hardwiring Excellence is a popular selling book [17], and was the topic of a presentation at the 2006 Association of University Programs in Health Administration.

### Searching

We conducted a review of the English language health, healthcare management, and organizational science literature (up to December 2007) for publications on each of these strategies. Each strategy was entered as a key word search in Medline, Web of Science, ABI/Inform, Cochrane Library, CINAHL, and ERIC. Results were limited to those dealing with U.S. health service organizations. Studies from other industries and foreign countries were not included. Our primary search resulted in 152 references on Six Sigma, 46 on Lean, and nine on StuderGroup's Hardwiring Excellence.

### Selection

Articles were included for review if they met the following five criteria: they appeared in a peer-reviewed journal; they described a specific intervention or activity prescribed by the transformation strategy; the intervention was not classified as a pilot study; they provided quantitative data describing the effect size or statistical significance; and they were not review articles. Peer-reviewed status was determined using publication information available in Ulrich's Periodicals Directory and the publication's website. These liberal criteria allowed for the inclusion of almost any study design, analytic strategy, outcome of interest, or type of health service organization. However, it served to exclude informational, tutorial, or advocacy pieces, news reports, and general success stories without sufficient data to critically judge the information presented.

After reviewing the titles, abstracts, and when necessary the full text according to the five review criteria, we included nine references on Six Sigma, nine on Lean/Toyota Production System, and one on StuderGroup for review. From each included article we abstracted a description of the intervention, the setting, study design, dependent variables, and key reported findings. The goal of this article was not to critique the interventions themselves, so the level of information extracted was not to the depth of very rigorous systematic comparative reviews such as a Cochrane EPOC review. Readers wishing to critically examine the interventions in greater detail are referred to the original publications.

### Data abstraction

Both authors reviewed the included studies and arrived at a consensus on the abstracted information. Setting included type of health service organization and if the article described an intervention within a hospital, the particular department in which the study occurred was noted.

## Results

Studies included in the review are summarized in Table S1; Additional File 1.

### Six Sigma

'Six Sigma is an organized and systematic method for strategic process improvements and new product and service development that relies on statistical methods and the scientific method to make dramatic reductions in customer defined defect rates' [18]. Motorola receives the credit for creating Six Sigma [19], but the methodology and concepts are clearly rooted in the quality improvement tradition promoted by Deming's TQM principles and the works of Juran [20,21]. Examining the methodology and philosophical underpinnings of Six Sigma are not an objective of this review, as Six Sigma's approach of problem identification, measurement, statistical analysis, improvement, and controls plans is well covered by numerous publications.

The nine studies included in this review described the results of Six Sigma programs on surgery turnaround time [22], clinic appointment access [23], hand hygiene compliance [24], antibiotic prophylaxis in surgery [25], scheduling radiology procedures [26], catheter-related bloodstream infections [27], meeting Centers for Medicare and Medicaid Services (CMS) cardiac indictors [28], nosocomial urinary tract infections [29], and operating room (OR) throughput [30]. While each study addressed a very different problem, they shared numerous common features. Bush and colleagues' report [23] on patient access was concerned with obstetrics and gynecological appointments at an outpatient clinic, while the remaining studies were set in various hospital departments. None were conducted by outside evaluators or researchers. While none of the studies were randomized trials, all included pre-intervention measurements. Also importantly, each reported their respective Six Sigma interventions were effective.

Parker and colleagues' [25] examination of an intervention to improve antibiotic prophylaxis during surgery reported statistically significant increases in the proportion of surgery patients receiving timely prophylaxis. However, methodological issues question these conclusions. Pre-intervention data were collected through retrospective chart review and post-intervention data were captured electronically during the procedure. Without a comparison group experiencing the same change in data collection, it is not possible to definitely exclude the change in measurement as responsible for the reported effect size. Additionally, while this study had the most sophisticated analysis of all the studies included on Six Sigma, the statistical inferences are biased. The authors compared pre- and post-intervention data using the X^2 ^statistic which requires independent observations. The data violated this assumption because individuals (anesthesiologists) contributed multiple observations. Even if the observations were independent, the selected univariate statistic could not account for any residual confounding bias. Finally, single setting interventions are obviously susceptible to limitations in generalizability to other settings.

The one group pre-test post-test design was also utilized by the studies on surgery case turnaround time [22], radiology scheduling [26], catheter-related blood stream infections [27], urinary tract infections [29], and OR throughput [30]. All of these studies reported positive results: patient-out to patient-in time was decreased [22], the variation in the number of telephone calls required to schedule procedures was reduced [26], there were less infections [27,29], and delays were reduced [30]. However, the limitations on these conclusions are very similar, and they are considered *en mass*, because they share so many limitations in common. The single group pre-test post-test design means factors outside the actual intervention cannot be excluded as reasons for the results. In particular, Adams and colleagues'[22], Volland's [26], Hansen's [29], and Fairbanks'[30] studies were all implemented with other improvement activities occurring concurrently in the organization. The individuals participating in these studies may have been exposed to other quality initiatives or messages. All five studies are also similar in that they did not engage any statistical tests for all outcomes, so no inferences can be made. Nor was there adjustment for potential confounding bias in any study. Finally, these interventions were specific to their respective protocols and environments, and may not be able to be replicated anywhere else. Additionally, the results may not be sustainable; this concern was evident in both the catheter-related infection article [27], and the urinary tract infection article [29]. Although neither were analyzed as an interrupted time series design, the authors nonetheless presented multiple post intervention observations that indicated multiple periods where rates returned to pre-invention levels.

Two of the Six Sigma studies also employing a single group pre-test post-test design are slightly different than the above and are worth noting separately. Eldridge and colleagues' study [24] on hand hygiene reported significant increases in compliance, and Elberfeld and colleagues' study [28] reported improvements in meeting CMS performance standards. Because both of these studies employ a nationally recognizable clinical guideline or standard, and were implemented across multiple sites, they are stronger than the other Six Sigma studies in terms of external validity. In spite of this strength, they still both share many of the same limitations and concerns, as noted above. In the case of the hand hygiene study [24], the authors do not specify what statistical method they employed. However, the unit of analysis was an observation of behavior and not an individual, so observations are again not independent, and the unspecified test would have to account for that correlation. Again like the above studies, this study design cannot exclude any historical event as a plausible alternative hypothesis. Another concern is attrition because the number of post-intervention sample size was 25% smaller. The story is again similar for the Elberfeld and colleagues' article [28] as indicators of appropriate patient care improved, but no comparison group was referenced, and statistical analysis was nonexistent.

In terms of strength of study designs, Bush and his colleagues' [23] study deserves special attention, since it was the only one of the nine to include a control group. Patients in the treatment clinic had to wait 30 fewer days for an outpatient obstetrics visit, patient time in the clinic decreased, gross revenue increased, and both initial and return visits increased. They compared similar measures collected during the same study period from an internal medicine outpatient clinic. The inclusion of the comparison group, which had no changes, strengthens the conclusion the intervention was the necessary and sufficient condition for the changes in outcomes. None of the other studies included a design which addressed the threat from outside events being responsible for any of their results. While benefiting from the stronger design, this study presented only descriptive statistics. No inferential statistics or multivariate analyses were conducted. The study had no adjustment for confounding bias or selection bias.

### Lean/Toyota Production System

Like Six Sigma, healthcare organizations adopted Lean system principles from manufacturing. Lean calls for cultural change and commitment and what have been called the 4-Ps – philosophy of adding value to customers, society, and associates; processes paying off over time; people and partners who are respected and developed; and problem-solving to drive organizational learning [31]. Much of the attention is focused specifically on work processes, quality, and efficiency.

The studies on Lean interventions meeting the inclusion criteria included interventions in hospital laboratories [32-36], a telemetry unit [37], a gynecologist and his associated cytology laboratory [38], intensive care units [39], and hospital-wide [40]. The majority of this group, however, routinely omitted statistical analysis, violated statistical test assumptions, failed to adjust for confounding, introduced selection bias, and through failure to include a comparison group cannot exclude other external events as potential sources of invalidity. For example, Bryant and Gulling's laboratory study [32] indicates Six Sigma was already in place before the Lean intervention was implemented. In addition, each study is limited in generalizability to a large degree when the interventions conducted under the auspices of Lean were very site specific. As an extreme example, while Raab, Andrew-JaJa and colleagues' study applied statistical testing and provided power calculations, it was essentially a sample of one 'single gynecologist who expressed enthusiasm about improving his Papanicolaou test sampling' [38]; therefore, suspecting a reactive effect, which limits external validity, is fairly logical. However, a couple of the studies bear further examination.

The surgical pathology laboratory of the Henry Ford hospital applied Lean principles in order to reduce any defect defined as 'flaws, imperfections, or deficiencies in specimen processing that required work to be delayed, stopped, or returned to the sender' [36]. The study also reported a statistically significant change in the distribution of effects, with post intervention effects occurring more frequently earlier in the process. The study provided power and sample sizes estimates and also selected a statistical test appropriate for the paired nature of the pre-test post-test observations on single laboratory staff. The study possessed many criteria for strong causal inferences: no ambiguous temporal sequence, no participant attrition, minimal threat of selection bias, and no changes in instrumentation. However, the single group pre-test post-test design cannot rule out the threat from history. Like many of the aforementioned studies, a comparison group or increased observation periods would have dramatically improved this study.

The results of Persoon and colleagues' analysis [34] of application of Lean principles to their chemistry laboratory allows for the discussion of two important points. Without explicit articulation, the study employed a single group interrupted time series design. While this study is susceptible to invalidity through history, the graphed data from the repeated nature of the design provides visual support of a causal inference because with the implementation of the intervention, the outcome measure changes direction. The outcome measure was a performance index created by the authors that was the percent of completions in a month minus the baseline target of 80%. This study illustrates why outcome measurements in these types of evaluation studies matter from both a statistical conclusion validity and generalizability perspective. By reducing each monthly metric by an absolute amount, the variation in each monthly measure was exaggerated when graphed and no statistical tests were performed. From a generalizablity perspective, novel outcome measures may have legitimate practical importance for the authors, but may be of less importance or difficult to translate to other settings. The results of this study also highlight the need for continued measurement beyond a single post-test measurement. While downplayed by the authors, the presented effect size of the intervention decayed and eventually disappeared over time.

Lastly, Furman and Caplan's examination of Lean at Virginia Mason Medical Center [40] warrants specific comment because it was an intervention on an actual Lean initiative at the system level. With the onset of Lean activities, the medical center established a patient safety alert system that allowed for reporting of events that threaten patient safety, and therefore provides opportunity for remediation. The actual outlined intervention was a series of specific changes to the alert system after two years of implementation in order to increase the number of reports, clarify classification, and provide staff support. The results of this single group interrupted time series design were an increase in the average number of reports and more employees, processes, and equipment removed from work until remedial plans were developed. While this study has sufficient statistical and design limitations to question the nature of its inferences, by presenting the intended organizational level deployment of Lean, the article stands as an interesting contrast to narrower applications in the reviewed articles.

### StuderGroup's Hardwiring Excellence

The StuderGroup's approaches and techniques gained notoriety through work with recent Baldrige Award-winning hospitals, which gives face validity to the transformation strategy. The intervention comes out of the socio-behavioral change arena by taking a customer-focused and employee-centered approach combined with organization-wide training and leadership behavior modeling to bring about significant cultural change and quality and financial gains. In contrast to the number of transformational strategies originating in manufacturing, StuderGroup's Hardwiring Excellence approach was created by a healthcare administrator.

A single multi-site study that implemented a StuderGroup intervention met the inclusion criteria for this review [41]. Using a pre-test post-test with control group design, the authors examine the effectiveness of nurse rounding, bedside visits to patients at regular intervals, on patient call light usage, patient satisfaction, and patient falls with forty-six units (medical, surgical, or combination) within a sample of 22 hospitals. The authors report a statistically significant reduction in call light use, increases in patient satisfaction scores for the intervention groups, and a reduction in falls. The study is generalizable to other hospitals given the use of a large number of hospitals of varying size and location, and the use of easily replicable treatments and outcomes. Finally, from the stronger study design, the study can make strong claims against any alternative hypothesis from history, testing, changes in instrumentation, regression, or maturation.

Despite these favorable points, several limitations prevent any firm conclusion that this study supports the effectiveness of StuderGroup's interventions. The analytic methods employed raise concerns over statistical conclusion validity because multivariate adjustments for confounding were absent and the analysis did not account for the correlated nature of the nested observations. Likewise, while the control group design is a stronger design strategy, the analytic strategy failed to capitalize on its benefits as data were analyzed without regard for the controls. Next, related to statistical concerns is the problem of selection bias. The authors rightly identify the potential for selection bias and the reality that any type of random assignment was not practical. However, randomization is not the only way to control for selection bias. Statistical and design options exist for addressing selection bias. Lastly, this study was only a single intervention within the larger scope of StuderGroup's recommendations and strategies. Even if the limitations of this study were overcome, it would only support the effectiveness of nurse rounding and not the entire StuderGroup strategy.

## Discussion

Very few studies in the literature meet the five inclusion requirements for this review, but those that did represented diverse applications of transformation strategies. While as individual studies none were particularly generalizable, the diverse settings and interventions of Six Sigma and Lean suggest, at least, these strategies are frequently employed in healthcare. The broad applicability of Six Sigma is similar to the wide applications of other statistical process controls [42], and the ability of each to be adapted to new settings should facilitate their rapid adoption [43]. As already noted, the study with the least concerns over external validity was the evaluation of the StuderGroup intervention. In addition, each of the reviewed studies concluded the respective interventions were effective, and more than one provided estimates of cost savings. For Lean and Six Sigma the effectiveness conclusion agrees with prior research in the manufacturing area. While a handful of the studies were methodologically stronger than others, all of the studies reviewed had significant threats to validity and were unable to rule out all alternative hypotheses. One might take some satisfaction from the fact that all of these studies attributed successes to the implementation of the various strategies. Unfortunately, the universally reported effectiveness of each strategy may also reflect a positive result publication bias [44].

Two immediate recommendations for research in transformation strategies suggested by this review are improvements in research methodologies and expansion of timeframes. Nearly all of the reviewed studies could be improved dramatically through more sophisticated statistical analysis or the addition of a comparison group. Large healthcare systems with multiple hospitals could execute stronger study designs with minimal additional effort, *e.g.*, a phase-in of interventions would allow later implementer sites to serve as controls for early implementer sites. Alternatively, if a comparison group is not readily feasible, the very nature of these interventions facilitates interrupted time series designs, as was reported in two of the studies. A well-executed time series design not only has stronger validity claims, but also allows for the examination of a sustained effect [45]. This latter design by nature encourages a longer time period for examination of effects. Kotter suggested organizational transformation as a process requires five to ten years to be fully realized [46]. If this long view of evaluation research is taken, necessarily intermediate measures of process increase in importance and relevancy. Also, the longer time period can offer additional evidence of sustainability.

Creative evaluation models are possible, too, in large systems where multiple transformational strategies and units of analysis are in play. Scalability of evaluation may increase, *i.e.*, be scaled up, division-wide and organization-wide, to aggregate impacts and interactions of multiple interventions. Alternatively, the evaluation may be scaled down to identify changes attributable to a specific intervention at smaller units. These methodological improvements could be facilitated with academic partnerships or through research trained administrators because industrial engineering departments are no longer widespread in hospitals [47].

While suggesting this avenue to improvement, we are aware that funding for evaluation and management research is not a priority for many health organizations. Again, however, this re-emphasizes the point for improved research studies in order to demonstrate the value of these strategies. The obvious potential for cost-savings or reductions were implied by the improvements in almost all of the reported studies, however, only a couple specifically indicated how much money was either saved [33] or how revenues were increased [23]. Justification for evaluation and research is made easier when expected savings are available to offset those costs and those savings are expected to be ongoing. Still other opportunities exist for improved partnering between health services researchers and practicing organizations. Academic medical centers represent innovative institutions with a history and expectation of research, thereby appearing to be natural settings for these types of investigations. Evaluation of these and other transformative strategies may be slightly different than historical interest in clinical applications, but through academic contacts industrial/system engineers are more accessible and the culture is still one of research. Additionally, those seeking executive health management degrees, student interns, or even professionals returning to school for advanced degrees while still employed all provide opportunities and interested individuals for collaboration.

Our interest is in gaining the maximum impact from the various strategies, a situation which is most likely to occur if some degree of fidelity is maintained in implementation. We are not suggesting that there is no value from less rigorous evaluation models, or even that useful insights cannot be derived from heuristically impressive results reported in other formats. But real understanding of 'what, how, and why' of what worked (or didn't), is unlikely to occur without more exacting research and evaluation standards. That is, evaluation strategies may benefit from a realistic perspective that seeks to better inform practitioners of the applied value of these efforts [48]. Given the substantial costs associated with these transformation strategies, healthcare managers seeking to adopt any strategy would be better served by demanding more exacting evaluation of the projects from their staff or consultants, or even better, include outside evaluators within the project budget. Organizational learning, like all learning, is based upon both action and reflection. Minimally evaluated innovations may still be successfully replicated in the same setting because of unspoken shared understandings; but chances of it working again at another site within the system or elsewhere may be very limited.

Returning to the conceptualization presented in Table 1, we suggested that transformation requires both changes in practice and culture. While all of three of the examined transformations advocate a cultural change, few of the reviewed studies examined indicators resembling organizational culture. The Lean patient alert system intervention provided limited data on culture in the form of patient safety culture, and the Six Sigma programs on surgery turnaround time and hand hygiene compliance reported staff satisfaction. However none of these studies, or the anecdotal evidence reported in other studies fully captures the multidimensional construct of organizational culture, leaving valid questions on these interventions' interaction with and affect on organizational culture unanswered.

The role of an organization's culture is not only important to safe healthcare delivery; it serves also as a precursor to other innovations [49]. A review of TQM applications to hospitals revealed the innovation frequently faces an adverse culture, and managers incorrectly assumed employees would automatically adhere to the new philosophy [50]. Specifically speaking about healthcare, Kovner and Rundall noted, '...efforts to introduce evidence-based decision making quickly wither and fade away because the organizational culture does not support evidence-based management' [51].

Lacking in the articles reviewed here, and maybe in their larger respective evaluations, is the extent to which such transformations are sustainable, and the extent to which the knowledge, attitudes, and skills developed from the transformation are retained and transferred to other problems and parts of the healthcare organization. The two exceptions to the question of sustainability are Furman and Caplan's report on the safety alert system at Virginia Mason Medical Center [40], which included more than four years of post-implementation observations, and Shannon and colleagues' nearly three-year study [39]. Some of the other reviewed studies reported measurements at one to two years post-implementation [23,27,28,33-36], but the rest were on much shorter timelines of a few months, reflecting the narrowly focused application of these strategies. Based upon the anticipated timeframe for transformation, noted above, it would be difficult to see or even expect widespread organizational transformation within these windows.

In addition, multiple transformation strategies can be implemented in concert. The integration of strategies was evident in this review. For example, Napoles and Quintana record consultant's Lean training program included Six Sigma instruction [33], and others noted how more than one transformative strategies was already in place within their organizations [22,26,29,30]. Likewise, while a predominately a cultural change strategy, StuderGroup emphasizes measurement and therefore efficiency change. The potential for interactions, synergies, appropriate sequencing, or even conflicts between different strategies raises practical questions amenable both to theoretical examination and empirical testing.

As stated above, this review was not exhaustive of all transformational interventions available to healthcare leaders. We did not examine TQM or CQI, as those have been the subject of previous reviews [52], or the additional healthcare specific strategies like application of the Malcolm Baldrige National Quality Award framework, LeapFrog Group initiatives or Institute for Healthcare Improvement programs. A similar critical review of these later strategies, particularly compared to the finding presented in this article, might prove to be particularly informative. Similarly, while our review was broad, it did not include the grey literature; as we stated we would not dismiss the potential for valuable insights from impressive results reported in other formats, but that area of reporting was not our main interest. Nor did our search strategy allow for the inclusion of studies involving individual components or particular methods of the above strategies conducted without their Six Sigma, Lean, or StuderGroup nameplates. As noted above, these strategies and approaches have roots in other disciplines and draw on other approaches and concepts, particularly the statistical control aspects, which have certainly been examined independently. However, our interest is in these proposed transformation strategies as complete packages, as that is how they are currently proposed to healthcare organizations.

Health systems are continually innovating. Required are transformational changes that fundamentally alter practices and culture for immediate improvements in care and ever increasing capacity for continuing improvement. Improving evaluation and understanding of the implementation and outcomes of such changes are essential to sustaining ongoing transformation and restricting any legacy of failure. The healthcare literature needs more reports of rigorous examinations of these transformation efforts and ongoing dialogue between the research and practice community addressing this critical topic.

## Competing interests

The authors declare that they have no competing interests.

## Authors' contributions

JV and LG conceived the research question for this review. JV carried out the database searching, abstracted information from included articles, interpreted the data, and prepared the manuscript. LG reviewed the included studies, arrived at consensus with the abstracted information, interpreted the data, and prepared the manuscript. Both authors read and approved the final manuscript.

## Author's information

JV is a health services research doctoral candidate and the project coordinator for the Center for Health Organization Transformation in the School of Rural Public Health at the Texas A&M Health Science Center in College Station, Texas. LG is Director of the National Science Foundation and industry supported Center for Health Organization Transformation and Professor and Head of the Department of Health Policy and Management in the School of Rural Public Health at the Texas A&M Health Science Center in College Station, Texas.

## Supplementary Material

Additional file 1 **Table S1**. Summaries of organizational transformation research in U.S healthcare by strategy. Click here for file
