# Supplementary material for: A critical review of the research literature on Six Sigma, Lean and StuderGroup's Hardwiring Excellence in the United States: the need to demonstrate and communicate the effectiveness of transformation strategies in healthcare
Source: Implement Sci. 2009 Jul 1;4:35. doi: 10.1186/1748-5908-4-35 (PMC2709888; doi:10.1186/1748-5908-4-35)
Supplement: Additional file 1 — Table S1. Summaries of organizational transformation research in U.S healthcare by strategy. [file 1748-5908-4-35-S1.doc]

**Additional file 1. Summaries of organizational transformation research in U.S healthcare** by strategy.

| **Study, Year** | **Setting** | **Research problem** | | | **Intervention** | **Dependent variables** | **Design** | **Reported key findings** |
| --- | --- | --- | --- | --- | --- | --- | --- | --- |
| *Six Sigma* | | | | | | | | |
| Adams *et al.* 2004 | Operating room in a single hospital | | Turnaround time in operating room detrimental to physician satisfaction and cost | | 1. Process mapping  2. Process redesign  3. New task assignments | A. Patient-out to patient-in time (minutes)  B. Surgeon-out to surgeon-in time (minutes) | Single group pre-test post-test | A. Patient-out to patient-in time decreased by seven minutes  B. Surgeon-out to surgeon-in time decreased by two minutes  C. Reduced variation and extreme events |
| Bush *et al.* 2007 | Obstetrics (OB) and gynecology (GYN) outpatient clinic at a single hospital | | Improve patient access to OB/GYN clinics | | 1. Changed resident scheduling  2. Added new clinic sessions  3. Hired 1.3 full-time equivalent nurse practitioner and certified nurse midwife  4. Procedure changes  5. Created weekly obstetric patient-only clinic  6. Culture change | A. Visit wait time (days)  B. Patient time in clinic (hours)  C. Initial visits  D. Return/repeat visits  E. Patient satisfaction  F. Gross clinical revenue | Pre-test post test with comparison group design | A. OB visit wait times decrease from 38  days to eight days  B. Patient time in clinic decreased 3.2 to 1.5 hours  C. Initial GYN visits increased 87% and OB increased 55%  D. Return GYN visits increased 66% and repeat OB visits increased 45%  E. Mean patient satisfaction increased  F. Gross revenue up 73% |
| Elberfeld *et al.* 2004 | Four hospital health system | | Performance on Centers for Medicare and Medicaid Services cardiac indicators | | 1. Education  2. Daily census to identify patients  3. Designated emergency department nurses as point persons  4. Protocol change  5. Reminder stickers  6. New discharge instruction sheets | A. β blocker administered within 24 hours of admission expressed as defect rate  B. ACE inhibitor at discharge for AMI patients expressed as defect rate | Single group pre-test post-test | A. and B. Meet all Centers for Medicare and Medicaid Services’ performance standards |
| Eldridge *et al.* 2006 | Intensive care units in 3 Veteran’s Affairs medical centers | | Increase compliance with hand hygiene recommendations | | 1. Process measurement  2. Alcohol based hand rub (ABHR) made available at the bedside and/or the entryway  to all patient rooms and antimicrobial soap at all sink  3. Staff education | A. Percent compliance  B. ABHR usage (mass) | Single group pre-test post-test | A. Observed compliance increased from 47% to 80%  B. ABHR usage increases were sustained for nine months |
| Fairbanks 2007 | Operating room in a single medical center | | Improving operating room throughput | | 1. Process measurement  2. Process mapping  3. Education  4. Introduced staging area for first cases of the day | A. Percentage of on-time starts  B. Turnaround times  C. Patient satisfaction | Single group pre-test post-test | A. Increase from 12% to 89%  B. Decrease in mean of 23.8 minutes to 17.9  C. Satisfaction on wait times, perceived employee team work and overall facility rating improved |
| Frankel *et al.* 2005 | Surgical intensive care unit in a single hospital | | Catheter-related bloodstream infections | | 1. Process measurement  2. Supervision by attending staff  3. Training  4. Materials made available  5. Protocol change including antibiotic-coated catheters for select patients | A. Catheter-related bloodstream infections infection rate  B. Number of catheters placed between catheter-related bloodstream infections | Single group pre-test post-test | A. Catheter-related bloodstream infections infection rate decreased from 11.0 to 1.7  B. Number of catheters placed between catheter-related bloodstream infections increased 650% |
| Hansen 2006 | Single regional medical center | | Reduce the rate of nosocomial urinary tract infections among inpatients | | 1. Chart review  2. Education  3. Free re-culturing  4. Laboratory protocol changes | A. Urinary tract infections per 1,000 patient days | Single group pre-test post-test | A. Rates within control |
| Parker *et al.* 2007 | Surgery units in a single hospital | | Inappropriately timed antimicrobial prophylaxis for noncardiac surgery patients | | 1. Process mapping  2. Training  3. Change of protocols  4. New data reporting system | A. Percentage of patients receiving antimicrobial prophylaxis within 60 minutes of incision  B. Interval in minutes between antibiotic administration and surgical incision | Single group pre-test post-test | A. Patients receiving antimicrobial prophylaxis within 60 minutes of incision increased from 38% to 86%  B. Time interval for antibiotic administration before surgical incision decreased from 88 to 38 minutes |
| Volland J. 2005 | Radiology depart-ment in a single hospital | | Number of phone calls necessary for clinics to schedule an appointment with radiology department was unsatisfactory | | 1. Hour changes  2. Procedure changes | A. Number of phone calls | Single group pre-test post-test | A. Average number of phone calls remained unchanged, but the variation (s.d. decreased from 1.0 to 0.5)  B. Reduced complaints about the process |
| *Lean/Toyota Production System* | | | | | | | | |
| Bryant and Gulling 2006 | Laboratory department in a single hospital | | | Eliminate waste and improve laboratory output | 1. Process redesign  2. Flow analysis | A. Collection-to-results time  B. Percent of results available by 7a.m. | Single group pre-test post-test | A. Collection-to-results time decreased from 65 to 40 minutes  B. Percent of results available by 7am decreased from 50% to 14% |
| Furman and Caplan 2007 | Medical center | | | Threats to patient safety not adequately reported | 1. Adaptation of existing patient safety alert reporting system to include more types of incidents and more detail  2. Added position to monitor and respond to alerts  3. Expanded 24-hour telephone line to include web enabled reporting | A. Average number of patient safety alerts per month  B. Average number of days to resolution  C. Number of employees taken offline  D. Number of processes/equipment taken off-line | Single group interrupted time series | A. Average number of patient safety alerts per month increased  B. No discernable Average number of days to resolution  C. Number of employees taken offline increased  D. Number of processes/equipment taken off-line increased |
| Napoles and Quintana 2006 | Laboratory department in a single hospital | | | Streamline operations for cost savings and improved turn around time | 1. Process redesign/batching  2. Staff training | A. Chemistry tests performed per full time employee  B. Hematology reports performed per full time employee  C. Cost savings | Single group pre-test post-test | A. Chemistry turn around time decreased from 160 minutes to 86 minutes  B. Hematology turn around time decreased from 103 minutes to 56 minutes  C. Reduced staff salaries by $489k and saved $37k in maintenance and supply costs |
| Nelson-Peterson and Leppa 2007 | Telemetry unit in single hospital | | | Improve efficiency by reducing waste and rework | 1. Rapid process improvement workshop focusing on workflow  2. Process redesign | A. Staff walking distance  B. Lead time (minutes to complete one cycle of workflow)  C. Percent of call lights on in a four-hour period  D. Percent of RN time spent in indirect/non-value-added care  E. Set up time (minutes for one cycle of care)  F. Nursing hours per patient day | Single group pre-test post-test | A. Staff walking distance decreased from 5,818 steps to 846  B. Lead time decreased from 240 to 126 minutes  C. Percent of call lights on down from 5.5% at baseline to 0%  D. Percent of RN time spent in indirect/non-value-added care decreased from 68% to 10%  E. Set up time decreased from 20 minutes to three minutes  F. Nursing hours per patient day decreased from 9.0 to 8.4 |
| Persoon *et al.* 2006 | Laboratory department in a single hospital | | | Improve chemistry turnaround time | 1. Processing mapping  2. One piece flow/process redesign | A. Performance index (points above or below 80% completion rate) | Single group interrupted time series | A. Performance index scores improved |
| Raab, Andrew-JaJa *et al.* 2006 | Single gynecologist and cytology laboratory | | | Improving Papanicolaou (Pap) test quality | 1. Checklist for each step in Pap test  2. Workflow process redesign | A. Test specimen adequacy  B. Error frequency  C. Frequency of undetermined significance category | Single group pre-test post-test | A. Decrease of 9.9% to 4.7% of inadequate Pap tests  B. Error frequency decreased from 9.2% to 7.8%  C. Decrease of 7.8% to 3.9% of tests in undetermined significance category |
| Raab, Grzybicki *et al.* 2006 | Cytology unit serving two hospitals | | | Diagnostic errors in thyroid gland fine-needle aspiration (FNA) | 1. Added intermediate interpretative service  2. Standardization of terminology | A. Sensitivity  B. Specificity  C. False-negative diagnoses  D. False-positive diagnoses  E. Non-interpretable rate  F. Surgery rate  G. Repeat FNA rate  H. Atypical rate | Single group pre-test post-test | A. No statistical change in sensitivity  B. No statistical change in specificity  C. No statistical change in false-negative diagnoses  D. No statistical change in false-positive diagnoses  E. Non-interpretable rate decreased from 19.8% to 7.8%  F. Surgery rate did not change statistically  G. Repeat FNA rate decreased 7.7% to 3.7%  H. No statistical change in atypical rate |
| Shannon *et al.* 2006 | Medical intensive care and coronary care units in a single hospital | | | Central line-associated bloodstream infections | 1. Staff education  2. Process and procedure redesign | A. Infection rate per 1,000 line days  B. Deaths  C. Number of lines placed per one infection occurence | Single group pre-test post-test with multiple post-test observations (per fiscal year) | A. Infections decreased from pre-intervention rate of 10.5 to 1.2, 1.6, and 0.4  B. The number of deaths decreased from 19 to 1, 2, and 0  C. The number of lines placed per one infection increased from 22 to 185, 135, and 633 |
| Zarbo *et al.* 2007 | Pathology laboratory in a single hospital | | | Defects in specimen processing causes delays, work stoppage or return to sender | 1. Practice standardization  2. *kanban* system implemented  3. established tracking log  4. Process improvements | A. Percent of defective cases  B. Distribution of defects by test phase | Single group pre-test post-test | A. Proportion of defects decreased from 27.9% to 12.5%  B. Proportion of defects found earlier in the test process increased |
| *Studer’s Hardwiring Excellence* | | | | | | | | |
| Meade *et al.* 2006 | Nursing units across 14 hospitals | Better patient-care management | | | Nurse conducted beside rounds (one- and two-hour interval intervention groups) | A. Patient call light frequency  B. Patient satisfaction  C. Number of patient falls | Pre-test post test with comparison group design | A. Reduction in total call light use for units with rounding  B. Increase in patient satisfaction scores  C. Reduction in falls for one hour rounding. |
